# Neuro‐Dynamic Quantitative Systems Pharmacology (Qsp) Model Supports Continued Lecanemab Treatment With Maintenance Dosing For Alzheimer’s Disease

**DOI:** 10.1002/alz.092093

**Published:** 2025-01-09

**Authors:** Youfang Cao, Brian A. Willis, Pallavi Sachdev, Natasha Penner, Kristin R Wildsmith, Arnaud Charil, Kanta Horie, Akihiko Koyama, Larisa Reyderman

**Affiliations:** ^1^ Eisai Inc., Nutley, NJ USA; ^2^ Eisai Co, Tsukuba Japan

## Abstract

A 10 mg/kg every 2 week (Q2W) dose of the humanized IgG1 monoclonal antibody lecanemab was approved after demonstrating significant clinical benefit in slowing cognitive decline in early Alzheimer’s disease (AD) in two clinical studies (the phase 2 Study 201 and phase 3 Clarity AD). A less frequent every 4 weeks lecanemab 10 mg/kg maintenance dosing (Q4W) has been proposed after a sufficient initial Q2W treatment. To further understand long‐term benefit of continued Q4W lecanemab treatment, a quantitative systems pharmacology (QSP) model was developed which mechanistically describes AD pathophysiology using multivariate data from clinical studies. The population QSP model incorporated 3 interlinked modules describing Aβ pathway, tau pathology and cognitive decline, where Aβ triggers tau pathology and tau pathology leads to cognitive decline due to neuronal damage and degeneration. The model was developed using nonlinear mixed‐effect modeling and was successfully validated using a multivariate dataset (N = 4057) consisting of amyloid PET, CDR‐SB, ADAS‐Cog14, plasma Aβ42/40 ratio and ptau181, and tau PET data from the lecanemab trials, as well as amyloid PET, ADAS‐Cog and CDR‐SB data from the Alzheimer’s Disease Neuroimaging Initiative (ADNI). Simulations were conducted to investigate long‐term lecanemab benefits with Q4W dosing following the initial lecanemab Q2W treatment. QSP simulations showed additional benefits gained across clinical outcomes, amyloid plaque and protofibrils, Tau PET, and plasma biomarkers with continued lecanemab treatment beyond 18 months. Specifically, maintenance lecanemab continued to reduce brain amyloid plaques and protofibrils in subjects who did not reach amyloid negativity following the initial 18‐month Q2W lecanemab. Simulations showed plasma ptau‐181 continued to decline and plasma Ab42/40 ratio continued to increase with Q4W maintenance dosing. Furthermore, simulations showed continued suppression of tau PET, and additional reductions in cognitive decline (CDR‐SB, ADAS‐Cog14) with Q4W maintenance dosing. Sustained protofibril clearance was indicated as a key driver for long‐term (>18 months) lecanemab effect on halting tau pathology and slowing cognitive decline, particularly after plaques are cleared following initial lecanemab Q2W treatment. QSP simulations demonstrated the importance of continuing lecanemab treatment after plaque removal and support Q4W maintenance dosing following initial treatment.

